# Affective Temperaments and Illness Severity in Patients with Bipolar Disorder

**DOI:** 10.3390/medicina57010054

**Published:** 2021-01-09

**Authors:** Mario Luciano, Luca Steardo, Gaia Sampogna, Vito Caivano, Carmen Ciampi, Valeria Del Vecchio, Arcangelo Di Cerbo, Vincenzo Giallonardo, Francesca Zinno, Pasquale De Fazio, Andrea Fiorillo

**Affiliations:** 1 Department of Psychiatry, University of Campania “L. Vanvitelli”, 80132 Naples, Italy; steardo@unicz.it (L.S.J.); gaia.sampogna@unicampania.it (G.S.); vito.caivano@gmail.com (V.C.); carmenciampi@gmail.com (C.C.); valeria.delvecchio78@gmail.com (V.D.V.); ardice77@gmail.com (A.D.C.); enzogiallo86@gmail.com (V.G.); francesca.zinno@yahoo.it (F.Z.); andrea.fiorillo@unicampania.it (A.F.); 2 Psychiatric Unit, Department of Health Sciences, University Magna Graecia, 88100 Catanzaro, Italy; defazio@unicz.it

**Keywords:** affective temperaments, bipolar disorder, illness severity, predictors

## Abstract

*Background and objectives:* Bipolar disorder (BD) is one of the most burdensome psychiatric illnesses, being associated with a negative long-term outcome and the highest suicide rate. Although affective temperaments can impact on BD long-term outcome, their role remains poorly investigated. The aims of the present study are to describe the clinical characteristics of patients with BD more frequently associated with the different affective temperaments and to assess the relation between affective temperaments and severity of clinical picture in a sample of patients with BD. *Materials and Methods:* A total of 199 patients have been recruited in the outpatients units of two university sites. Patients’ psychiatric symptoms, affective temperaments, and quality of life were investigated through validated assessment instruments. *Results:* Predominant cyclothymic and irritable temperaments are associated to higher number of relapses, poorer quality of life, higher rates of aggressive behaviors, and suicide attempts. Conversely, the predominant hyperthymic disposition was a protective factor for several outcome measures, including relapse rate, severity of anxiety, depressive and manic symptoms, suicidality, and earlier age at onset. One limitation of the present study is that the recruitment took place in two university sites; therefore, our findings cannot be fully generalized to the whole community of BD patients. Other limitations are the lack of a control group and the cross-sectional design of the study. *Conclusions:* The early identification of affective temperaments can help clinicians to identify those BD patients who are more likely to show a poor long-term outcome. An early screening of affective temperaments can be useful to develop targeted integrated pharmacological and psychosocial interventions.

## 1. Introduction

Bipolar disorder (BD) is a highly disruptive and chronic illness affecting between 0.3 and 1.5% of individuals worldwide [1]. Compared to any type of cancer or major neurological diseases, BD is responsible for the loss of more disability-adjusted life years (DALYs), which are mostly due to early onset and chronic course [2,3]. Among psychiatric disorders, BD is associated with the highest suicidal rate, with about 33% of patients affected by BD showing a history of suicide attempts [1].

BDs are currently categorized in well-defined diagnostic categories, which are operationalized by the Diagnostic and Statistical Manual for Mental Disorders – Fifth Edition (DSM-5) and the International classification of Diseases – 11^th^ Version (ICD-11) diagnostic manuals into discrete entities [4]. However, the heterogeneity of the clinical picture of bipolar disorders in terms of symptomatology, treatment response, and needs for care, as well as social, family and biological correlates, represents a challenge to the adoption of well-defined diagnostic criteria in clinical practice [5,6]. In fact, the adoption of a broad bipolar spectrum has been proposed, with the identification of several different clinical phenotypes [7].

According to the concept of the bipolar spectrum, the assessment of affective temperaments and the clinical characterization of their association with psychiatric symptoms can contribute to explaining the heterogeneity of mood episodes, to identify predictive factors of good or poor outcome, and to select appropriate treatments [8].

The term “temperaments” refers to the emotional domain of personality [9], and can be considered the interface between biological and psychological features of affective disorders. Temperaments are defined as a stable trait of personality and refer to patients’ activity levels, social and biological rhythms, mood disposition, and daily variability [10,11]. The first definition of affective temperaments was provided by Kraepelin, who described the depressive, irritable, manic, and cyclothymic dispositions [12] and conceptualized these traits as fundamental states of episodes of severe depression and/or mania. Kretschmer [13] unified the different fundamental Kraepelinian states to the broad concept of cyclothymia and schizothymia, which were considered as normal variants of temperament. Mental disorders, whether affective or psychotic, were considered as exaggerated forms of normal temperamental traits. Psychopathic variants (named ‘cycloidia’ and ‘schizoidia,’ respectively) were considered by Kretschmer as kinds of intermediate states between temperamental types and related disorders. On the basis of the early definitions provided by Kraepelin and Kretschmer, Akiskal [10] has developed the modern theoretical conceptualization of affective temperaments by describing the following five temperaments: cyclothymic, depressive, hyperthymic, irritable, and anxious. The cyclothymic temperament presents continuous and sudden fluctuations of mood and levels of energy, between mild hypomanic/irritable and mild depressive/dysphoric phases; the depressive temperament is characterized by the persistence of a dysthymic trait, and a tendency to a lowered mood; the anxious temperament is defined by the presence of a stable state of excessive anxiety and worries; the irritable temperament is characterized by impulsivity and anger as core traits, whereas the hyperthymic temperament consists of stable high levels of energy, optimism, self-confidence, and elevated mood [14,15].

The different temperamental dispositions can be considered a subclinical (trait-related) manifestation of bipolar disorders that significantly contribute to their clinical course, including the direction of polarity, symptom profiles, and severity of affective episodes [16]. The studies on temperaments have contributed to the definition of the boundaries of bipolar spectrum and to increasing the scientific knowledge on the relationship between affective temperaments and the “classical” affective symptoms [17].

According to the available studies, cyclothymic temperament is often associated with young age at onset, high number of affective episodes, psychotic symptoms during acute phases [18,19], and comorbidity with anxiety disorders, in particular panic/agoraphobia and social anxiety disorder. Moreover, cyclothymic and irritable temperaments are highly linked with impulsivity and aggressiveness, and therefore, patients with these dispositions often present aggressive behaviors and suicide attempts [19]. The hyperthymic disposition is characterized by more severe manic episodes requiring hospitalization [20].

In this study, we aim to describe the clinical characteristics of BD more frequently associated with affective temperaments, and to explore which affective temperaments are associated with a more severe clinical picture and a worse long-term outcome of bipolar disorder. We hypothesized that several clinical indicators, including age at onset, number of affective episodes, and lifetime suicidal attempts are strongly correlated with the five affective temperaments proposed by Akiskal. In particular, we anticipate that the cyclothymic and irritable affective dispositions are associated with worse clinical picture and long-term outcome of the disorder.

## 2. Methods

The study was carried out at the Mood Disorder outpatient Units of the Department of Psychiatry of the University of Campania “Luigi Vanvitelli” and of University Magna Graecia of Catanzaro. All referring patients between June 2019 and January 2020 were included in the study if they met the following inclusion criteria: (1) diagnosis of type-I or type-II bipolar disorder, confirmed by the Structured Clinical Interview for DSM-5 disorders; (2) age between 18 and 65 years; (3) willingness to participate in the study, expressed by written informed consent provided upon complete description of the protocol; (4) being in a stable phase of the disorder since at least 6 months. Exclusion criteria were: (1) inability to give a written consent to participate in the study; (2) diagnosis of any neurological disease; (3) presence of actual drug and/or alcohol abuse, according to the DSM-5. The study was carried out in accordance with the latest version of the Declaration of Helsinki and was approved by the Local Research Ethic Committee (Number: N001567/28.01.2018).

## 3. Procedures and Measures

### 3.1. Socio-Demographics Characteristics

Patients’ socio-demographic (i.e., gender, age at study entry, employment, and educational level) and clinical characteristics (i.e., age at onset, lifetime number of affective episodes, pattern of illness course, presence of mixed affective states, number of suicidal attempts, and alcohol and drug misuse) were recorded with an ad hoc schedule. Previous affective episodes have been defined according DSM-5 criteria. Alcohol misuse was defined as consuming eight or more drinks per week for women, or 15 or more drinks per week for men [21]. Drug misuse was defined as the daily use of any substance (including cannabis, cocaine, opiates, hypnotics, stimulants, hallucinogens, and solvents) for at least two weeks in any period of life. Patients with substance abuse disorders, according to DSM-5 criteria, have been excluded from the study. On the contrary, patients with an occasional use of alcohol or substances during an acute phase of the disorder have been included, in order to have a better and more real characterization of enrolled patients.

### 3.2. Psychopathological Assessments

Depressive and anxiety symptoms were assessed through the 17-item Hamilton Depression Rating Scale (HAM-D) [22] and the 14-item Hamilton Rating Scale for Anxiety (HAM-A) [23], respectively. Manic symptoms were assessed with the Young Mania Rating Scale (YMRS) [24], and the quality of life with the Manchester Short Assessment of Quality of Life (MANSA) [25]. Affective temperaments were assessed through the Italian short version of the Munster Temperament Evaluation of the Memphis, Pisa, Paris, and San Diego (bTEMPS-M). The bTEMPS-M is a 35-item version developed by Erfurth et al. [26] on the basis of the TEMPS-A, originally developed by Akiskal et al. [27]; the main change from the original version is the scoring of items, which are now on a 5 point Likert-type scale (1 = “not at all”; 2 = “a little”; 3 = “moderately”; 4 = “much”; 5 = “very much”), while it was a yes/no answer in the Akiskal original version. The aim of this change was to improve the clinical and research utility of the scale allowing for an exploration of the dimensionality of the investigated domains. The factor analysis confirmed the five-factor structure of the original scale, including the cyclothymic, depressive, irritable, hyperthymic, and anxious subscales. The Italian 35-item TEMPS-M has shown good psychometric properties in terms of reliability and validity and it is characterized by a dimensional structure, supporting the concept of the bipolar spectrum, with a Cronbach’s alpha of the five subscales ranging from 0.898 to 0.808 [28].

### 3.3. Statistical Analyses

Descriptive statistics were calculated for socio-demographic and clinical characteristics, as well as for scores of relevant assessment instruments. Data were presented as means (M) and standard deviations (SD), or as frequencies and percentages (%), as appropriate. The Kolmogorov–Smirnov test was used to check the normality of distribution of our sample.

We identified several socio-demographic and clinical characteristics that, on the basis of the existing literature, could be considered valid proxies for worse outcome, including age at onset, duration of untreated illness, total number of hospitalizations, total number of affective episodes, presence of aggressive behaviors, psychotic symptoms, and suicide attempts [1,29,30]. Correlation analyses were performed to assess the association between affective temperaments and the clinical and socio-demographic characteristics expressed as continuous variables. The *t*-Student test for independent samples was performed to assess the association of affective temperaments with clinical and socio-demographic characteristics expressed as discrete variables. According to the independent variables, linear and logistic regression models were performed to test whether affective temperaments can be considered predictors of outcome. These models were corrected for diagnoses, age, sex, and pharmacological treatments. The level of statistical significance was set at *p* < 0.05. Statistical Package for Social Sciences version 21 was used to perform the statistical analyses.

## 4. Results

### 4.1. Socio-Demographics and Clinical Characteristics

A total of 199 patients have been recruited (Table 1). Half of the sample (50.8%) is female, with a mean age of 47.1 ± 13.2 years. Of the recruited patients, 54.8% have a primary diagnosis of bipolar I disorder, with a mean age at onset of 27.0 ± 9.5 years. Additionally, 42.3% of patients has psychotic symptoms during affective episodes, 25.6% used drugs and/or alcohol during an affective episode, 56.8% reported at least one episode of aggressive behaviors, and 30.2% has at least one suicidal attempt. The mean number of affective episodes is 11.0 ± 10.4. The mean scores at bTEMPS-M are 22.6 ± 6.7 for the depressive subscale, 19.1 ± 6.1 for the hyperthymic one, 18.9 ± 6.2 for the anxious subscale, 23.3 ± 7.6 for the cyclothymic subscale, and 18.9 ± 8.1 for the irritable one. Finally, patients have a mean score of 4.7 ± 7.2 at HAM-A, 8.0 ± 10.8 at HAM-D, 4.6 ± 8.4 at YMRS, and 46.7 ± 11.4 at MANSA.

### 4.2. Univariate Analyses

Pearson’s correlations and *t*-Student analyses are reported in Table 2 and Table 3, respectively. The depressive temperament is positively correlated with the total number of hospitalizations (*p* < 0.0001) and with the total number of affective episodes (*p* < 0.01), and negatively with the MANSA total score (*p* < 0.0001).

The hyperthymic temperament is positively correlated with age of onset (*p* < 0.01), and negatively with the total number of hospitalizations (*p* < 0.001), the YMRS total score (*p* < 0.01), and with the HAM-D total score (*p* < 0.05).

Anxious temperament positively correlated with the HAM-D total score (*p* < 0.01), and negatively with MANSA total score (*p* < 0.01).

Cyclothymic temperament showed a positive correlation with the total number of hospitalizations, HAM-A (*p* < 0.01), and HAM-D total scores (*p* < 0.05), while it is negatively correlated with MANSA total score (*p* < 0.0001).

Lastly, irritable temperament positively correlated with HAM-A and HAM-D total scores, with the total number of affective episodes (*p* < 0.001), with YMRS total score (*p* < 0.01), and with the total number of hospitalizations (*p* < 0.05), and negatively correlated with the MANSA total score (*p* < 0.0001).

**Figure 1:**
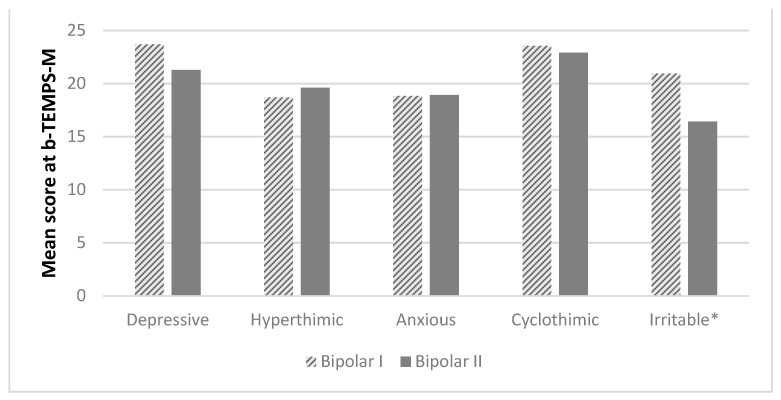
Differences in affective temperaments according to diagnosis. * *p* < 0.0001. bTEMPS-M = The Italian short version of the Munster Temperament Evaluation of the Memphis, Pisa, Paris, and San Diego.

The distribution of the five affective temperaments in bipolar I and bipolar II disorders shows that patient with BD I have a cyclothymic or irritable disposition more frequently compared with patients with BD II, although this difference is statistically significant only for the irritable disposition (*p* < 0.0001) (Figure 1).

Aggressive behaviors are more frequent in patients with cyclothymic, depressive (*p* < 0.01), or irritable (*p* < 0.001) temperaments. Psychotic symptoms are more frequently reported by patients with a predominant depressive or irritable (*p* < 0.0001) disposition. Substance misuse is reported only by patients with a hyperthymic temperament (*p* < 0.01), while suicide attempts are present in all temperamental dispositions, with the exception of the hyperthymic one, which seems to have an inverse association.

### 4.3. Multivariate Analyses

According to the linear and logistic regression models (Table 4 and Table 5), having a predominant cyclothymic temperament increases the risk of being hospitalized (*p* < 0.05), having a worse quality of life (*p* < 0.05), more aggressive behaviors (*p* < 0.01), and more suicide attempts (*p* < 0.01). The predominant irritable temperament increases the risk of having more severe depressive symptoms (*p* < 0.05), a worse quality of life (*p* < 0.01), more aggressive behaviors (*p* < 0.01), more psychotic symptoms (*p* < 0.01), and more suicide attempts (*p* < 0.01). Patients with a predominant hyperthymic temperament have a later age at onset (*p* < 0.05), a reduced risk of being hospitalized (*p* < 0.01), less severe anxiety, depressive and manic symptoms (*p* < 0.05), and less frequent suicide attempts (*p* < 0.01), with an increased probability of substance abuse (*p* < 0.05). The anxious and the depressive temperaments are not associated with any of the considered outcome measures. 

## 5. Discussion

The correlations between affective temperaments and long-term outcome of BD is an emerging theme. Most studies previously carried out on this topic have investigated the impact of single affective temperaments on single outcome measures, usually the number of relapses or hospitalization, the presence of drug abuse, suicide attempts, aggressive behaviors, and the severity of affective symptoms, but a comprehensive analysis on the relationship between the five affective temperaments and several outcome measures in BD is missing.

The main finding of our study is that temperaments influence the clinical severity of bipolar disorder. In particular, patients with predominant cyclothymic and irritable temperaments have a higher number of relapses, a reduced quality of life, higher rates of aggressive behaviors, and suicide attempts. On the contrary, the hyperthymic disposition seems to be a protective factor against clinical severity, being associated with a lower relapse rate, less severe anxiety, depressive and manic symptoms, reduced suicidality, and earlier age at onset. Moreover, patients with an hyperthymic temperament presented drug misuse more frequently during affective episodes compared with the other four affective temperaments.

These data are consistent with available literature and show that the hyperthymic temperament is orthogonally different from all the other affective dispositions [20]. The hyperthymic disposition is mainly characterized by mood-emotional intensity and high level of energy, while the other four temperaments could be grouped into a single disposition, mainly characterized by emotional instability and rapid fluctuations of mood. Although this distribution was already known [31,32], its clinical implications and impact on the long-term outcome of BD were not clear.

Another clinically relevant finding is that the hyperthymic temperament is associated with a better symptom profile and less suicide attempts [33]. This finding can be due to the fact that the hyperthymic temperament is associated with an increased level of energy, ambition, drive, confidence, cheerfulness, social skills, optimistic attitude toward life, and increased creativity [14]. BD patients with an hyperthymic temperament may have attenuated symptoms and a better adaptation to environmental stressors [33] due to more pronounced resilience skills, problem-solving strategies, and a general better attitude toward life adversities [34]. These personal resources could be at the core of the protective role of this temperament.

Notably, our results show that the hyperthymic disposition increases the likelihood to present drug misuse in patients with BD. In a previous study, Singh et al. [35] found that alcohol abuse was significantly predicted by the hyperthymic and irritable dispositions. The comorbidity between substance abuse and BD is associated with an earlier onset of mood symptoms, more relapses, increased suicide risk, more violent behaviors, more hospitalizations, and more frequent treatment non-adherence [36]. According to the Cloninger’s temperament dimensions [37], this finding can be due to the negative association of hyperthymic disposition with harm avoidance, and to the positive association with novelty seeking. High levels of novelty seeking are associated with increased alcohol use, while low levels of harm avoidance may cause a reduced knowledge about the negative consequences of dangerous behaviors. However, this finding should be cautiously considered, since patients with a comorbid substance abuse were not included in our study, but the presence of substance misuse was considered as a proxy of impulsive behaviors.

Another significant finding is the strong relationship of cyclothymic and irritable temperaments with the severity of clinical symptoms. In fact, those patients present more severe anxiety and depressive symptoms, a higher number of relapses and hospitalizations, more frequently psychotic symptoms, more aggressive behaviors, and suicide attempts. Most of these associations have been confirmed at the multivariate analyses, showing that patients with predominant cyclothymic or irritable dispositions have a highly recurrent course of illness, with a pattern of impulsiveness, suicidality, and reduced quality of life. This finding may be related to the high mood lability and emotional overactivity, as well as to impulsive–aggressive emotionally erratic behaviors and hypersensitivity that are characteristic of these two affective dispositions [38]. Moreover, these affective dispositions have been identified as early predictors and risk factors for a variety of mental disorders, including bipolar, anxiety, and depressive disorders, thus indicating their relevance in influencing the clinical profile of patients with severe mental disorders [20].

Our results also showed the presence of a considerable overlap between the cyclothymic and irritable temperaments in terms of psychopathological characteristics, thus confirming that these two dispositions operate simultaneously rather than being two different dimensions [39]. Future research should examine the biological and psychological processes behind cyclothymic and irritable temperaments and should clarify whether they are independent, overlapping, or subordinate constructs.

Although several outcome measures correlate with depressive (i.e., number of hospitalizations, total number of affective episodes, quality of life, aggressive behaviors, and presence of psychotic symptoms during acute phases) and anxious (HAM-D total score and quality of life) temperaments, none of these correlations is confirmed at the regression analyses. These findings confirm that the depressive and anxiety temperaments cannot be considered strong predictors of poor outcome of patients with bipolar disorder [9,15,20,34]. It may be possible that the presence of depressive and anxiety predominant temperaments influence the clinical presentation of BD, including its polarity, but have no influence on illness severity.

The following limitations should be acknowledged. First, given the limited sample size and the fact that the recruitment took place in two university sites only, our findings cannot be fully generalized to the whole community of patients with bipolar disorder. Second, there is a lack of a control group made by patients affected by another mental disorder; therefore, we could not clarify if the impact of the affective dispositions on clinical severity and long-term outcome is disease-specific or can be applicable to other mental disorders. Another limitation is the fact that the correlation between affective temperaments and the presence of other psychiatric comorbidities was not considered. However, all comorbidities will be analyzed in further analyses from our dataset. Last, the cross-sectional design of the study did not allow us for multiple assessments. Although affective temperaments can be considered stable traits, multiple assessments can provide a more accurate and ecological assessment of outcome measures associated with affective dispositions.

In conclusion, our results highlight that the different affective temperaments have a different predictive role on clinical severity and outcome of bipolar disorder. In particular, while the cyclothymic and irritable temperaments seem to be risk factor for a worse outcome, the hyperthymic temperament has a more protective role. Since affective temperaments are considered innate, enduring, and stable traits, an early assessment of temperaments could help to identify those BD patients who are more likely to present a more severe long-term course and outcome. An early screening of affective temperaments can be used to develop personalized and integrated early pharmacological and psychosocial interventions to patients with bipolar disorder, according to the precision medicine approach.

## Figures and Tables

**Table 1:** Socio-demographic and clinical characteristics of the sample.

	Global Sample (N = 199)
Age (M ± DS)	47.10 ± 13.19
Gender, Male, % (N)	49.2 (98)
Years of education (M ± DS)	13.32 ± 3.46
Age at onset (M ± DS)	26.98 ± 9.47
Total number of hospitalizations (M ± DS)	0.68 ± 0.94
Years lived with the disorder (M ± DS)	20.01 ± 12.43
Number of affective episodes (M ± DS)	10.97 ± 10.37
Presence of suicide attempt, % (N)	30.2 (60)
Number of suicide attempts (M ± DS)	0.42 ± 0.88
Predominant polarity, % (N)	
Manic/hypomanic	10.6 (21)
Depressive	14.1 (28)
Undifferentiated	75.4 (150)
Depressive affective temperament (M ± DS)	22.61 ± 6.74
Hyperthymic affective temperament (M ± DS)	19.13 ± 6.14
Anxious affective temperament (M ± DS)	18.90 ± 6.22
Cyclothymic affective temperament (M ± DS)	23.28 ± 7.56
Irritable affective temperament (M ± DS)	18.91 ± 8.06
HAM-A, total score (M ± DS)	4.73 ± 7.24
HAM-D, total score (M ± DS)	7.97 ± 10.80
MRS, total score (M ± DS)	4.65 ± 8.43
MANSA, total score (M ± DS)	46.67 ± 11.44
History of aggressive behaviors, yes, % (N)	56.8 (113)
Presence of psychotic symptoms during acute phases, yes, % (N)	42.3 (80)
Treatment with lithium, yes, % (N)	50.8 (95)
Positive family history for psychiatric disorder, % (N)	69.8 (88)
Drug misuse, % (N)	25.6 (51)

M = Mean; DS = Standard Deviation; HAM-A = Hamilton Anxiety Rating Scale; HAM-D = Hamilton Depression Rating Scale; MRS = Mania Rating Scale; MANSA = Manchester Short Assessment of Quality of Life.

**Table 2:** Correlations between affective temperaments and clinical characteristics.

Affective Temperament	Age at Onset	Total Number of Hospitalizations	Total Number of Episodes	HAM-A, Total Score	HAM-D, Total Score	MRS, Total Score	MANSA, Total Score
Depressive	−0.058	0.352 ****	0.174 **	0.133	0.121	0.122	−0.457 ****
Hyperthymic	0.157 **	−0.248 ***	−0.064	−0.125	−0.148 *	−0.181 **	0.123
Anxious	−0.098	0.118	0.123	0.086	0.180 **	0.115	−0.301 **
Cyclothymic	−0.103	0.214 **	0.072	0.165 **	0.154 *	0.098	−0.366 ****
Irritable	−0.083	0.172 *	0.197 ***	0.190 ***	0.193 ***	0.165 **	−0.447 ****

* *p* < 0.05; ** *p* < 0.01; *** *p* < 0.001; **** *p* < 0.0001; HAM-A = Hamilton Anxiety Rating Scale; HAM-D = Hamilton Depression Rating Scale; MRS = Mania Rating Scale; MANSA = Manchester Short Assessment of Quality of Life.

**Table 3:** Comparison between affective temperaments and clinical characteristics.

	Aggressive Behaviors		Psychotic Symptoms during Acute Phases		Suicide Attempts		Substance Misuse	
	Yes	No	Yes	No	Yes	No	Yes	No
Depressive	23.48 ± 6.73	21.48 ± 6.63 **	24.49 ± 6.61	21.20 ± 6.67 ***	25.27 ± 6.46	21.47 ± 6.56 ****	22.10 ± 5.745	22.79 ± 7.066
Hyperthymic	18.81 ± 6.44	19.55 ± 5.74	18.46 ± 5.73	19.41 ± 6.33	17.00 ± 6.03	20.05 ± 5.98 ***	20.88 ± 5.99	18.53 ± 6.09 **
Anxious	19.61 ± 6.37	17.97 ± 5.92	19.44 ± 6.66	18.37 ± 5.95	21.30 ± 6.64	17.86 ± 5.75 ***	19.06 ± 6.12	18.84 ± 6.27
Cyclothymic	24.27 ± 7.48	21.98 ± 7.51 **	23.71 ± 7.50	22.63 ± 7.70	26.32 ± 6.54	21.96 ± 7.61 ****	23.14 ± 7.79	23.32 ± 7.51
Irritable	20.56 ± 8.13	16.76 ± 7.46 ***	22.04 ± 8.57	16.59 ± 6.79 ****	21.52 ± 8.51	17.79 ± 7.61 **	20.22 ± 7.59	18.47 ± 8.19

** *p* < 0.01; *** *p* < 0.001; **** *p* < 0.0001; Data are expressed as means ± standard deviations.

**Table 4:** Linear regression analyses.

	Total Number of Hospitalizations		Age at Onset		HAM-A. Total Score		HAM-D. Total Score		MRS. Total Score		MANSA. Total Score	
	B (95% CI)	P	B (95% CI)	P	B (95% CI)	P	B (95% CI)	P	B (95% CI)	P	B (95% CI)	P
Depressive	0.02 (−0.005 to 0.046)	0.119	0.146 (−0.106 to 0.398)	0.256	−0.039 (−0.251 to 0.174)	0.719	0.214 (−0.566 to 0.137)	0.231	−0.058 (−0.035 to 0.189)	0.643	−0.275 (−0.632 to 0.081)	0.129
Hyperthymic	**−0.030 (−0.053 to −0.008)**	**0.009**	**0.237 (0.027 to 0.448)**	**0.027**	**−0.185 (−0.363 to −0.007)**	**0.041**	**−0.332 (−0.626 to −0.038)**	**0.027**	**−0.263 (−0.469 to −0.057)**	**0.013**	0.200 (−0.129 to 0.529)	0.231
Anxious	−0.026 (−0.054 to 0.001)	0.062	−0.142 (−0.391 to 0.107)	0.262	−0.76 (−0.286 to 0.135)	0.479	0.187 (−0.161 to 0.535)	0.291	0.048 (−0.196 to 0.292)	0.699	0.059 (−0.316 to 0.435)	0.755
Cyclothymic	**0.027 (0.005 to 0.050)**	**0.017**	−0.122 (−0.332 to 0.088)	0.255	0.162 (−0.015 to 0.339)	0.073	0.178 (−0.115 to 0.471)	0.233	0.068 (−0.137 to 0.274)	0.512	**−0.329 (−0.640 to −0.018)**	**0.038**
Irritable	−0.015 (−0.034 to 0.003)	0.100	−0.052 (−0.219 to 0.115)	0.539	0.160 (0.019 to 0.302)	0.260	**0.255 (0.022 to 0.488)**	**0.032**	0.0156 (−0.008 to 0.319)	0.062	**−0.421 (−0.677 to −0.165)**	**0.002**
Constant	−0.057 (−0.859 to 0.754)	0.887	0.299 (0.208 to 0.390)	0.000	3.122 (−3.589 to 9.833)	0.360	0.161 (0.034 to 0.288)	0.013	−0.047 (−0.136 to 0.043)	0.304	−0.110 (−0.236 to 0.017)	0.088

B = Beta coefficient; P = *p*-value; HAM-D = Hamilton Depression Rating Scale; MRS = Mania Rating Scale; MANSA = Manchester Short Assessment of Quality of Life. Linear regression analysis with “Total number of affective episode” is not reported since no predictors have been found. Statistically significant results are highlighted in bold.

**Table 5:** Logistic regression analyses.

	Aggressive Behaviors		Psychotic Symptoms		Suicide Attempts		Substance Abuse	
	B (95% CI)	P	B (95% CI)	P	B (95% CI)	P	B (95% CI)	P
Depressive	−0.004 (0.935 to 1.061)	0.897	1.102 (0.93–1.30)	0.254	−0.28 (0.904 to 1.046)	0.446	−0.015 (0.916 to 1.061)	0.699
Hyperthymic	−0.018 (0.931 to 1.036)	0.503	0.973 (0.85–1.11)	0.686	**−0.096 (0.851 to 0.970)**	**0.004**	**0.080 (1.017 to 1.152)**	**0.013**
Anxious	0.021 (0.959 to 1.088)	0.517	0.981 (0.84–1.15)	0.815	0.039 (0.967 to 1.118)	0.293	0.053 (0.980 to 1.135)	0.156
Cyclothymic	**0.021 (0.968 to 1.078)**	**0.008**	0.980 (0.86–1.11)	0.747	**0.77 (1.016 to 1.149)**	**0.002**	−0.027 (917 to 1.033)	0.379
Irritable	**0.041 (0.998 to 1.087)**	**0.004**	**1.147 (1.03–1.28)**	**0.002**	**0.040 (0.994 to 1.090)**	**0.008**	0.001/0.956 to 1.049)	0.952
Constant	−0.889	0.384	1.102	0.254	−3.842	0.006	−2.580	0.031

B = Beta coefficient; P = *p*-value; Statistically significant results are highlighted in bold.

## Data Availability

The data that support the findings of this study are available on request from the corresponding author. The data are not publicly available due to privacy or ethical restrictions.
